# Biomechanical Evaluation of Cantilevered 2-Unit Implant-Supported Prostheses: A 3D Finite Element Study

**DOI:** 10.1016/j.identj.2025.01.014

**Published:** 2025-02-11

**Authors:** Hatem S. Sadek, Noha M. Anany, Al-Hassan Diab, Mohamed I. El-Anwar, Abdulaziz Alhotan, Mostafa Aldesoki, Christoph Bourauel, Tarek M. Elshazly

**Affiliations:** a Oral Technology, Dental School, University Hospital Bonn, Bonn, Germany; b Department of Operative Dentistry, Faculty of Dentistry, Ain shams University, Cairo, Egypt; c Department of Oral Medicine, Periodontology and Diagnosis, Faculty of Dentistry, British University in Egypt, Cairo, Egypt; d Department of Mechanical Engineering, National Research Centre, Giza, Egypt; e Department of Dental Health, College of Applied Medical Sciences, King Saud University, Riyadh, Saudi Arabia

**Keywords:** Prosthodontic, Zirconia, PEKK, CAD/CAM, FEM, Stress analysis

## Abstract

**Objective:**

To assess the biomechanical performance of cantilevered 2-unit implant-supported prostheses with zirconia and polyetherketoneketone (PEKK) under 3 loading conditions.

**Method:**

A cone beam computer tomography (CBCT) scan of an edentulous mandible was segmented using Mimics software and refined in 3-Matic software to create trabecular and cortical bone structures. Implant CAD files were integrated using SolidWorks software, generating 4 models with varying implant positions: M1 (first premolar implant supporting a second premolar), M2 (second premolar implant supporting a first premolar), M3 (second premolar implant supporting a first molar), and M4 (first molar implant supporting a second premolar). Prostheses were constructed from zirconia or PEKK. Finite element analysis (FEA) in ANSYS software simulated static loading: vertical (100 N) and oblique (30° and 45°, 50 N). von Mises stress and total deformation were analyzed.

**Results:**

Vertical loading showed the highest von Mises stress at cantilever connectors, with M3 displaying the highest cortical bone stress (117 MPa). Zirconia models had slightly higher prosthetic stress, while PEKK models exhibited greater implant and cortical bone stress. Oblique loading caused higher stress in implants and prostheses but remained below yield limits. Maximum deformation was under 25 micrometers for the implant and bone, and 65 micrometers for the prosthesis.

**Conclusion:**

Single implants can support 2-unit cantilevered prostheses when additional implants are unfeasible. The location of the cantilever has minimal impact compared to its size, as a larger cantilevered part increases stress. Zirconia better resists bending forces and reduces implant stress compared to PEKK.

**Clinical significance:**

This study guides prosthodontists in designing 2-unit implant-supported prostheses, emphasizing that multiple implants optimize stress distribution, and that zirconia is preferable for cantilevered designs.

## Introduction

Nowadays, implant therapy consistently demonstrates high success rates and effectively addresses many challenges faced by edentulous patients, significantly improving their quality of life.^1^, ^2^, ^3^ Nonetheless, various biomechanical factors have been found to influence the longevity and effectiveness of implant treatment, including the quality and quantity of edentulous bone tissue, the number and distribution of inserted implants, the design of the prosthetic superstructure, as well as the type of patient occlusion and masticatory forces.^1^^,^^4^^,^^5^

Adequate bone volume is a crucial prerequisite for optimal implant placement. However, the bone resorption that follows tooth loss may complicate this process.^6^ Insufficient bone volume with no possibility for bone augmentation, or failure of a previously inserted implant to osseointegrate, may prohibit standard implant placement. Additionally, the limited mesiodistal space in certain clinical scenarios may restrict the placement of the required number of implants.^7^ In such cases, cantilevered implant-supported fixed prostheses may serve as an alternative treatment option to circumvent more invasive surgical procedures requiring additional time and cost and entail a higher risk of postoperative complications.^8^^,^^9^

However, clinical studies^10^, ^11^, ^12^ have reported greater marginal bone loss at the implant-bone interface nearest to the cantilever arm, likely due to excessive loading. Hence, understanding the biomechanical behavior of different cantilevered implant configurations is crucial for optimizing load distribution, minimizing stress, and preventing bone resorption that could compromise implant survival and longevity.^1^^,^^13^ Additionally, selecting suitable prosthetic materials for implant-supported prostheses remains a challenging aspect of such treatment planning.^9^^,^^14^ Modern dental implant prostheses use advanced biomaterials to enhance success and patient outcomes. Titanium is the gold standard, while zirconia offers esthetic, metal-free options. PEEK reduces stress shielding with bone-like elasticity, and Co-Cr alloys and ceramic-metal hybrids provide durability and cost-effective options.^15^, ^16^, ^17^

Finite element analysis (FEA) is a widely used method for assessing the biomechanical performance of implants across various clinical scenarios.^7^^,^^18^ It is an advanced engineering tool that enables structural analysis of bodies with complex geometries and varying material properties by dividing the structure into a finite number of elements connected by nodes. With an appropriate mesh and mathematical model, FEA can simulate structural reactions and interactions.^19^ FEA serves as a reliable tool for estimating stress distribution patterns on prosthetic components, the implant system, and peri-implant bone.^7^^,^^20^ This analysis can aid in selecting the most appropriate design and materials for each clinical case, optimizing outcomes, as well as enhancing implant longevity.^21^^,^^22^

The aim of the current study was to evaluate the biomechanical behavior of a 2-unit implant-supported prosthesis with different configurations in the posterior mandible, in order to address the knowledge gap arising from the limited number of studies on this topic. The evaluation was conducted using 2 different prosthetic materials under vertical and oblique loading conditions, analyzed by FEA. The first null hypothesis posited that the cantilever position (mesial or distal) would not affect the biomechanical behavior of the 2-unit implant-supported prosthesis. The second null hypothesis stated that variations in prosthetic material would not lead to differences in stress distribution within the implant-prosthetic assembly.

## Methods

### Study design

In this study, 4 identical FEA mandibular models were created. In each model, only 1 implant was placed in different locations supporting a 2-unit prosthesis with different prosthetic designs as described in Table 1. The implant-supported prostheses were modeled in 2 different prosthetic materials: monolithic zirconia (Z) and Polyetherketoneketone (PEKK) (P). Hence, the models created were as follows: 4 models for zirconia (M1-Z, M2-Z, M3-Z, M4-Z) and 4 models for PEKK (M1-P, M2-P, M3-P, M4-P).

**Table 1:** Model designs based on positioning of implant and cantilevered crown.

Model	Implant position	Cantilevered crown	Symbol
Model 1 (M1)	First premolar	Second premolar	4+C5
Model 2 (M2)	Second premolar	First premolar	5+C4
Model 3 (M3)	Second premolar	First molar	5+C6
Model 4 (M4)	First molar	Second premolar	6+C5

### Geometric model creation

**Fig. 1:**
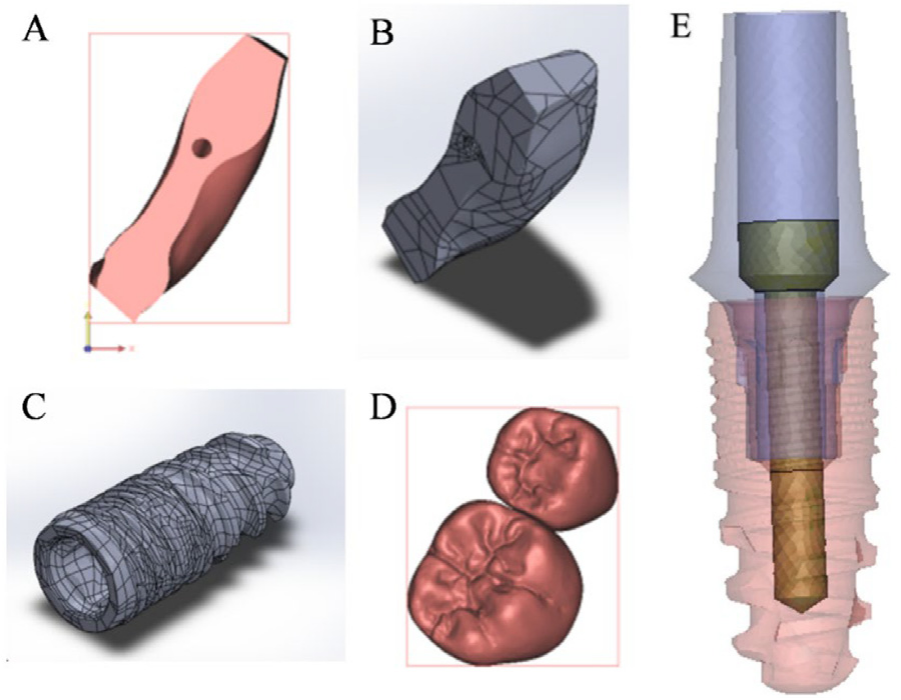
Components of the model: A, Compact bone, B, Spongy bone, C, Implant fixture, D, Two-units prothesis, E, Implants complex (abutments, screws, fixtures).

Following approval from the Ethics in Research Committee at the Faculty of Dentistry, Ain Shams University, Egypt (approval number FDASU-ReclE121904), a cone-beam computed tomography (CBCT) scan of an edentulous mandible was processed using Mimics 14 software (Materialise, Leuven, Belgium). The segmented data was exported as a Standard Tessellation Language (STL) file and imported into 3-Matic 7.01 software (Materialise, Leuven, Belgium) for further refinement. In 3-Matic, the mandible model was inspected for geometric errors, smoothed, and modified to include a trabecular bone core surrounded by a 2 mm-thick cortical shell, generated through a uniform offset operation. Additionally, a 1 mm-thick mucosal layer was modeled over the superior surface of the mandible. Subsequently, the CAD models of a dental implant system (Dentaurum, Ispringen, Germany), consisting of a fixture (9.0 mm length, 3.7 mm diameter), abutment, and fixing screw, were imported as STL files for integration in the model. Four models were designed as mentioned above and exported into Exocad DentalCAD software (Exocad, Darmstadt, Germany) to create the suprastructure (Prothesis) (Figure 1).

The geometry of these solid components was then exported as an IGES file to engineering CAD/CAM software (SolidWorks V. 2014; Dassault Systèmes, Aix-en-Provence, France) to correct any errors that may have occurred during the transformation from the point cloud to solid geometry. Subsequently, the solid parts of the model components were exported as a STEP file for processing in finite element software (ANSYS; Canonsburg, PA, USA). Finally, Boolean operations were performed within ANSYS to verify the validity of contact surfaces and finalize the geometric models. The solid components of the geometric models are shown in Figure 1. The materials were assumed to be isotropic, linear, and elastic. The material parameters of each component are listed in Table 2.

**Table 2:** Properties of the experimental materials used in the finite element models.

Material	Modulus of elasticity [GPa]	Poisson's ratio	Reference
Prosthesis 1: monolithic zirconia	200.00	0.26	Beuer et al.^23^
Prosthesis 2: polyetherketoneketone	5.10	0.40	Alqurashi et al.^24^
Implant complex	110.00	0.34	De Moor et al.^25^
Mucosa (soft tissue)	10.00	0.40	Abaza et al.^26^
Cortical bone	13.70	0.30	Al-Zordk et al.^27^
Cancellous bone	1.37	0.30	Al-Zordk et al.^27^

### Mesh formation

**Fig. 2:**
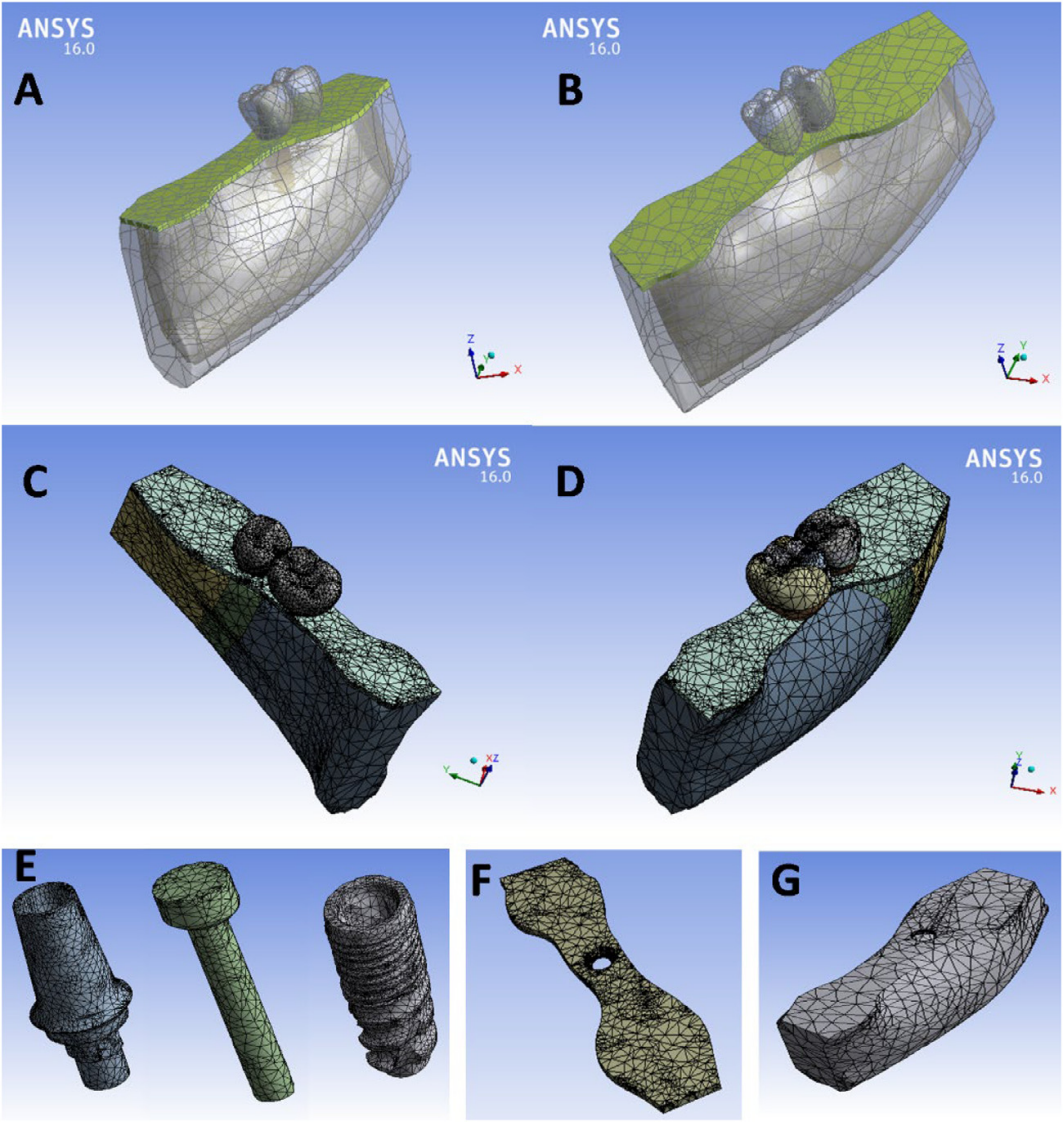
Components of the finite element model: A, Model #1, B, Model #2, C, Meshed model #3, D, Meshed model #4, E, Implant complex (abutment, screw, fixture), F, Mucosa, and G, Bone.

The solid geometries of the 4 models were discretized into a finite number of elements using 3D brick solid elements (Element type 187).^28^ A mesh convergence test was conducted by applying test loads across different mesh densities to ensure the accuracy of the results for the experimental models. This test determined the minimum number of elements required to ensure reliable numerical results. It involved applying loads to models with varying mesh densities to evaluate result accuracy, ensuring consistency within approximately ±5% variation in von Mises stress on the cortical bone. Convergence testing demonstrated that a smooth transition with a growth rate of 1.2 achieved convergence, with element sizes ranging from 0.005 mm to 0.991 mm. This range effectively captured the complex details of the models. As a result, each model's mesh was generated with varying numbers of elements and nodes, as outlined in Table 3. Screenshots of the meshed components, generated in ANSYS, are presented in Figure 2.

**Table 3:** Mesh density of the 4 models' components.

	Model 1: 4+C5		Model 2: 5+C4		Model 3: 5+C6		Model 4: 6+C5	
Component	Elements	Nodes	Elements	Nodes	Elements	Nodes	Elements	Nodes
Prosthesis	5434	8787	4295	7625	4533	7914	5130	8492
Abutment	20,655	35,465	20,400	35,868	20,490	35,972	20,914	36,278
Screw	3941	6936	3807	6750	3013	6,338	4089	7888
Implant	27,082	46,794	27,757	47,779	27,574	47,482	27,440	47,463
Mucosa	8676	16,113	7716	14,501	7440	14,001	8812	16,229
Cortical bone	16,626	31,015	16,504	30,991	16,288	30,092	16,783	31,304
Cancellous bone	26,875	41,773	26,235	41,546	25,126	41,002	26,901	41,793

### Contact conditions, boundary conditions, and loading protocol

Glue contact was assigned to the interfaces between different model components, assuming complete osseointegration between the bone and implant, as well as the occurrence of cold welding at the implant component interfaces.^29^ As boundary conditions, the mesial and distal planes of the bone volume were fixed in all 3 directions to prevent movement during load application. Before conducting the numerical analysis, the models were validated against similar studies.^23^^,^^27^ Each model underwent 3 different loading scenarios on the functional cusp(s) of the cantilevered crown, resulting in a total of 24 case studies. The first scenario involved applying a 100 N vertical load, the second used a 50 N oblique load at a 30° angle, and the third involved a 50 N oblique load at a 45° angle. Linear static analyses were performed on a high-performance workstation (HP Z820; HP, Palo Alto, CA, USA) equipped with Dual Intel Xeon E5-2660 processors (2.2 GHz) and 64 GB RAM. The von Mises stresses and the maximum total deformation across various components of the models were calculated.

### Statistical analysis

FE simulations yield deterministic and repeatable results for a given set of input parameters, as they are governed by predefined mathematical models and boundary conditions. Unlike experimental studies that are subject to variability and randomness, FE outputs are fixed for identical inputs. As a result, statistical metrics like mean and standard deviation, which require variability across data points, are not applicable to FE studies. Instead, sensitivity analysis, as described in the mesh formation section, is more appropriate for assessing the influence of varying parameters in FE models.^19^

## Results

**Fig. 3:**
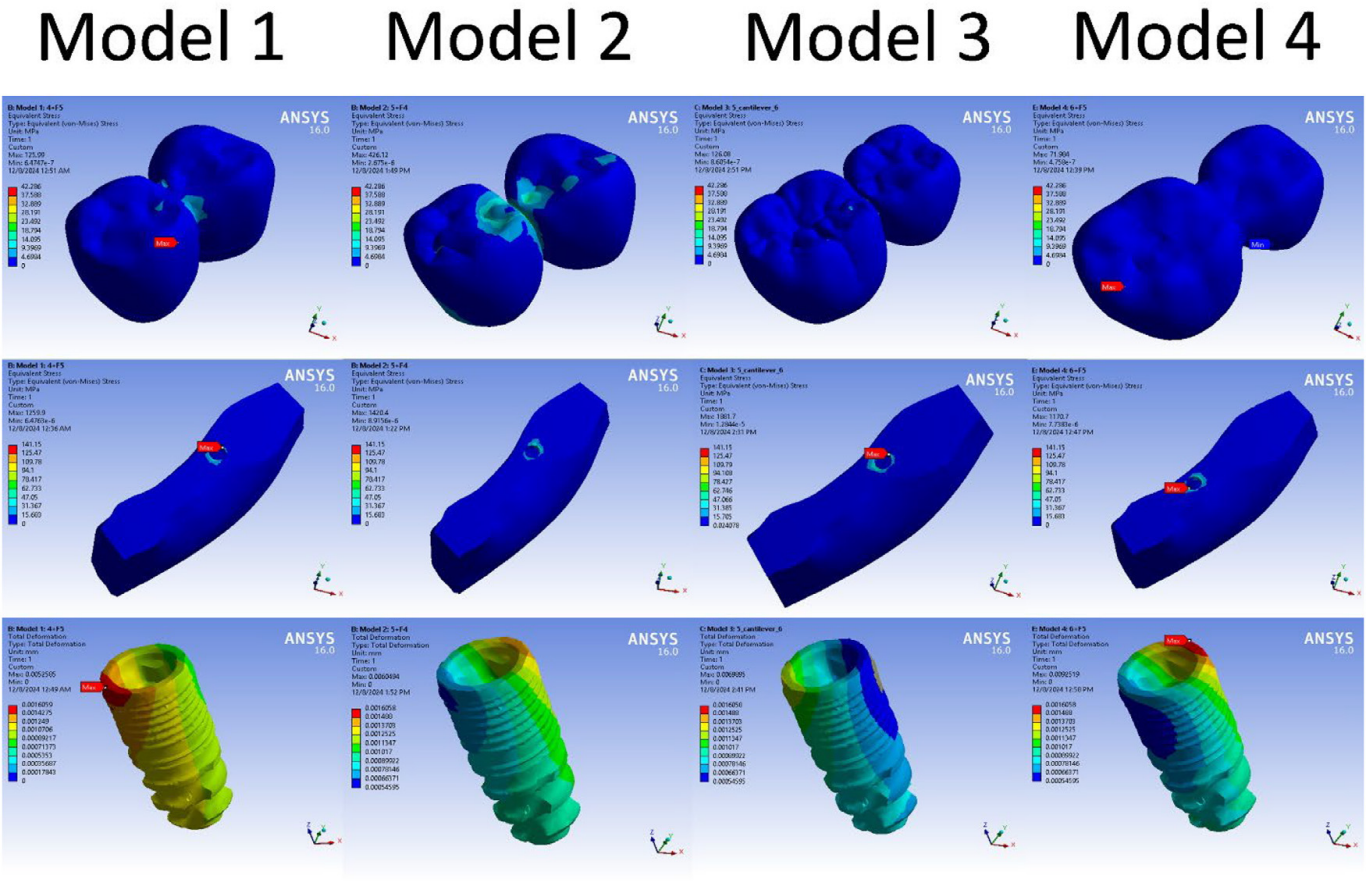
Color-coded screenshots of the vertical loading scenario showing equivalent von Mises Stress distribution in the prosthetic body (Top), and Bone (Middle), as well as the total deformation in the implant (Bottom).

The FEA results demonstrated stress distribution patterns across each component of the implant-prosthetic assembly under 3 loading scenarios (Figure 3). In the vertical loading scenario, the highest von Mises stress values were concentrated at the connectors of the cantilevered crowns (Figure 3). Model 3 (5+C6) exhibited the highest von Mises stress values at the cortical bone (117 MPa), while models 1 and 2 displayed only minor differences in stress values. The monolithic zirconia models showed slightly higher stress values in the prosthetic body compared to the PEKK models, while PEKK models exhibited slightly higher stress values at the implant and cortical bone.

**Fig. 4:**
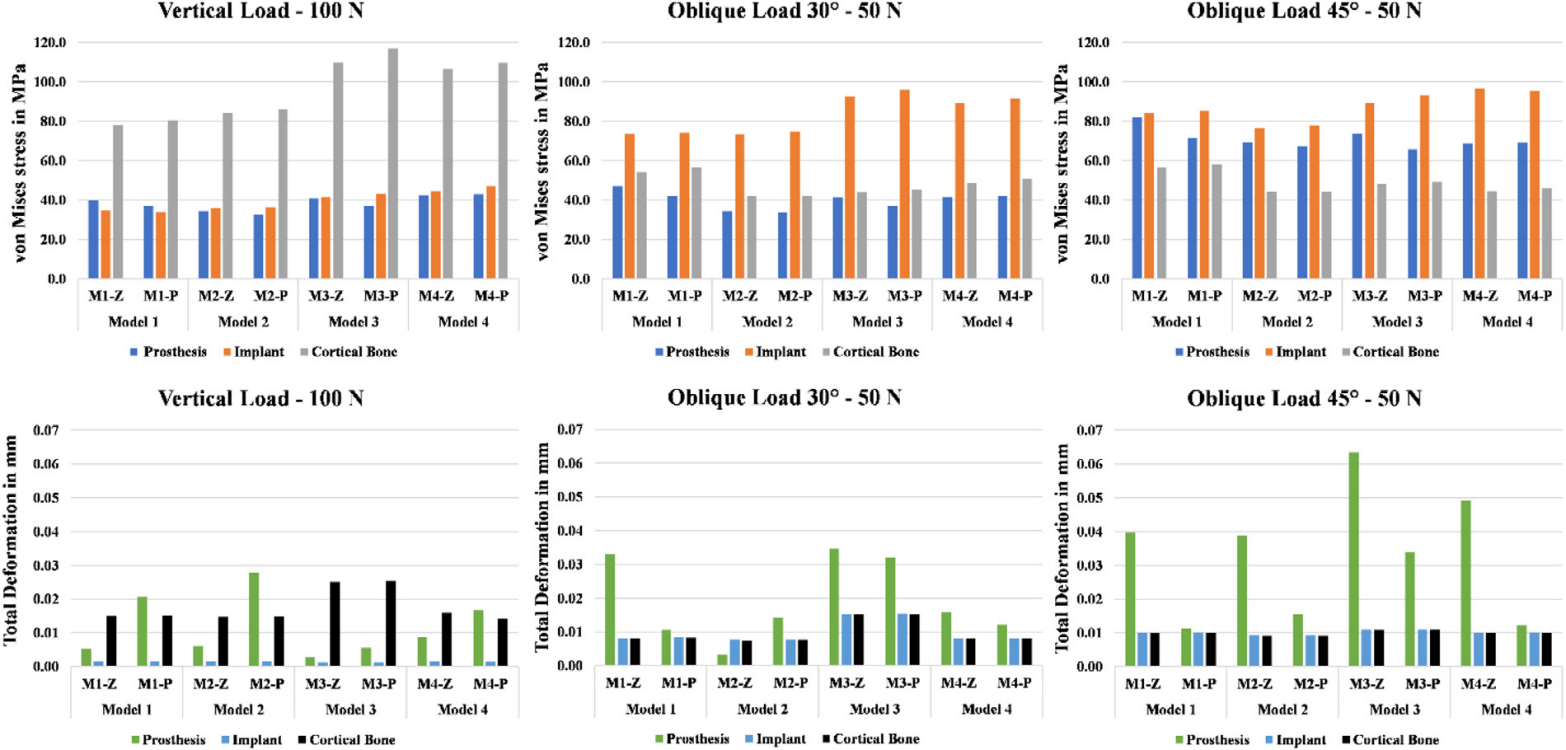
Equivalent von Mises stress and maximum total deformation results for various components of the finite element model, comparing different designs, materials, and loading scenarios.

Under the 30º oblique loading scenario, the stress distribution resembled that of the vertical loading condition, with Model 3 continuing to show the highest von Mises stress values at the implant (Figure 4). However, reducing the load to 50 N decreased the stress exerted on the bone relative to the 100 N vertical loading. In contrast to the bone, the implant and prosthesis exhibited higher stress values under oblique loading than under vertical loading, though all values were well below the yield stress of the tested materials, indicating no risk of failure.

The 45º oblique loading scenario produced results similar to those of the 30º oblique loading, with higher stress values observed in the implant and prosthesis compared to vertical loading. The monolithic zirconia models continued to show slightly higher stress values in the prosthetic body and lower stress values in the implant and cortical bone than the PEKK models (Figure 4). Stress values for zirconia remained within a safe range, while PEKK values approached its yield stress.

Maximum total deformation for the implant and cortical bone across all 4 models did not exceed 25 micrometres under both vertical and oblique loading conditions (Figure 4). For the prosthetic body, the highest deformation was observed in M3-Z model, with values below 65 micrometres. All deformation values remained within acceptable limits.

## Discussion

The use of a single implant to support a cantilever extension has become a viable solution, particularly in clinical scenarios where space is limited or anatomical constraints make the placement of a second implant challenging, often requiring invasive surgery with potential post-operative complications.^9^^,^^11^ Despite its growing relevance, the available reports in the literature on cantilevered implant-supported prostheses for partially dentate patients remain deficient. To address this gap, several consequent studies^7^^,^^17^^,^^30^ were conducted to evaluate various designs and materials for such prostheses.

In the current study, the biomechanical performance of different configurations of cantilevered single implant-supported 2-unit prostheses was assessed. The results demonstrated that cantilever configurations significantly influenced stress distribution patterns in the posterior mandible, leading to the rejection of the first null hypothesis. Moreover, differences in prosthetic materials with varying elastic properties showed a notable impact on stress distribution within the whole system, supporting the rejection of the second null hypothesis.

Cantilever extensions in prostheses are associated with increased risks of mechanical complications.^17^ Common complications, according to Romeo and Storelli,^31^ include veneer fractures (10%), screw loosening (7.9%), crown de-cementation (6.9%), and abutment screw fractures (1.6%). Palmer et al.^11^ and Wiskott et al.^32^ noted higher incidences of screw loosening in 2-unit cantilever prostheses compared to single-tooth replacements due to increased bending moments and rotational forces. Rangert et al.^33^ reported that cantilever designs elevate bending overload on implants, potentially compromising prosthesis prognosis. Nevertheless, all stress levels observed in the current analysis remained within physiological limits,^34^ with no indications of failure. This aligns with the reported survival rates of cantilever prostheses in the literature, despite some variability. Zurdo et al.^35^ reported a 5-year survival rate of 72% for prostheses with cantilever extensions versus 86% without, while Roccuzzo et al.^9^ documented a 100% implant survival rate for anterior 2-unit cantilevers over 33 months. Palmer et al.^11^ and Halg et al.^12^ found comparable survival rates of cantilevered and non-cantilevered prostheses, with stress levels remaining within the bone's load tolerance.

In the current study, mesial and distal cantilevers in the premolar region (M1 and M2) demonstrated similar stress distributions, consistent with previous findings.^9^^,^^11^ Romeo et al.^36^ recorded in one study no significant differences in marginal bone resorption between mesial and distal cantilevers, though mesial cantilevers had a slightly lower success rate (97.1%) compared to distal ones (100%). While in another study by Romeo et al.,^37^ it was concluded that the prognosis of implant-supported prostheses and marginal bone loss was unaffected by the position or length of the cantilever extension. They emphasized that proper control of factors such as cantilever length, prosthesis function, and occlusion is essential for achieving a favourable medium- to long-term prognosis for implant-supported prostheses.

Stress differences between premolar-premolar cantilevers were smaller compared to molar-premolar configurations, aligning with Aboelfadl et al.,^30^ who attributed higher forces to the larger occlusal table and the increased distance between the implant axis and the load application line, resulting in higher bending moment and rotational forces. Hence, M3, where the mandibular first molar acted as a distal cantilever crown, expressed the highest von Mises stress value among all study models. This comes in agreement with Alshiddi et al.,^17^ who reported that longer distal cantilevers significantly reduce the fracture load of implant-supported zirconia prostheses, highlighting the importance of minimizing their length for longevity. Besides, the connector area exhibited the highest von Mises stresses across all cases, likely due to increased bending forces at this site.^1^^,^^38^ Therefore, Alshiddi et al.,^17^ reported also that reinforcement of the connector area by increasing the thickness of the distal abutment significantly increases the fracture load of the implant-supported zirconia prostheses.

Vertical occlusal forces are well-tolerated by implants and natural teeth, as they align with the tooth axis and cause minimal shear stresses. In contrast, oblique forces, though of similar magnitude, create higher shear stresses and bending moments, increasing the risk of implant failure, prosthesis fracture, and bone resorption.^39^ Therefore, minimizing oblique loading is crucial for the longevity and success of dental implants. In the current study, stress values under oblique loading were higher than those under vertical loading, even when the load magnitude was halved, consistent with Batista et al.^1^ This can be referred to the decomposition of oblique load energy into vertical and lateral components, with the lateral component introducing additional shear and torque on the prosthesis-implant complex. Furthermore, Takahashi et al.^40^ showed that oblique loading increased von Mises stresses and shifted stress concentrations to the trabecular bone near the implant apex and platform. Nonetheless, all stress levels in the cortical bone and implant remained well below fatigue limits.^34^

Regarding prosthetic material, zirconia exhibited slightly higher stress within the prosthetic body and lower stress in the implant and cortical bone compared to PEKK, consistent with prior studies.^30^^,^^41^^,^^42^ This is due to zirconia's rigidity, which concentrates stress within the prosthetic structure while minimizing stress transmission to the implant and bone, enhancing implant longevity. In contrast, PEKK, with its lower elastic modulus, acts as a shock absorber but causes greater deformation and higher stress at the implant-bone interface.^30^^,^^41^^,^^42^ Rigid zirconia is well-suited for cantilevered designs to withstand bending forces and reduce implant stress, while resilient PEKK shows promise for fixed-fixed designs with lower bending forces.^43^

By having a close insight and comparing the results of M3 in the current study (1 implant at lower 5 supporting a 2-unit prosthesis with a distal cantilever at lower 6) to a previous design in another study^44^ (2 implants at lower 4 and 5 supporting a 3-unit prosthesis with a distal cantilever at lower 6) revealed higher von Mises stresses in the single-implant model under both vertical and oblique loading for both tested materials (monolithic zirconia and PEKK). Similarly, M2 (1 implant at lower 5 supporting a 2-unit prosthesis with a mesial cantilever at lower 4) showed higher stress values compared to a prior design with 2 implants at lower 5 and 6 supporting a 3-unit prosthesis with a mesial cantilever at lower 5. These findings indicate that increasing the number of supporting implants optimizes stress distribution and overall biomechanical performance, regardless of cantilever position. Moreover, it is worth noting that the effect of material difference was more significant in cantilevered long-span implant-supported prostheses compared to 2-unit implant-supported prostheses.

This study has several limitations. It was assumed that all analysed structures were homogeneous, isotropic, and linearly elastic simplifications deviating from clinical reality. Additionally, the loading scenarios did not fully capture the complexity of actual clinical conditions, omitting critical factors such as masticatory muscle activity and jawbone flexibility, all of which significantly influence load distribution and stress. Moreover, the study was limited to 2-unit implant-supported prostheses in the lower premolar-molar region, without exploring variations in cantilever length, implant location, or implant and prosthesis designs. Future research should employ more precise experimental setups and clinically relevant models while conducting experimental and clinical studies to validate these numerical findings and comprehensively assess the performance and prognosis of diverse prosthetic materials and designs.

## Conclusions

Within the limitations of this study, the following conclusions can be drawn:

• A single implant supporting a 2-unit cantilevered prosthesis in the posterior mandible is a viable treatment option when multiple implant placement is not feasible.
• Increasing the number of implants supporting a cantilevered prosthesis improves stress distribution and overall biomechanical performance.
• Larger cantilevered areas generate higher stresses at critical regions within the prosthetic system.
• The position of the cantilevered segment, whether mesial or distal, has minimal biomechanical impact when cantilevered teeth are of similar size.
• Rigid monolithic zirconia is more suitable for cantilevered designs due to its ability to withstand bending forces and minimize implant stress, compared to the more resilient PEKK.

## Declaration of Generative AI and AI-assisted technologies in the writing process

During the preparation of this work, the authors used the ChatGPT AI tool to improve the readability of the English language. After using this tool, the authors reviewed and edited the content as needed and took full responsibility for the content of the publication.

## CRediT authorship contribution statement

**Hatem Sadek:** Methodology, Investigation, Data curation, Validation, Writing – review & editing. **Noha Anany:** Methodology, Investigation, Data curation, Validation, Writing – original draft, Writing – review & editing. **Al-Hassan Diab:** Writing – review & editing. **Mohamed I. El-Anwar:** Methodology, Investigation, Data curation, Validation, Writing – review & editing. **Abdulaziz Alhotan:** Writing – review & editing. **Mostafa Aldesoki:** Methodology, Investigation, Data curation, Validation, Writing – review & editing. **Christoph Bourauel:** Methodology, Investigation, Data curation, Validation, Resources, Writing – review & editing. **Tarek Elshazly:** Methodology, Investigation, Data curation, Validation, Resources, Writing – original draft, Writing – review & editing. All authors have read and agreed to the published version of the manuscript.

## Compliance with ethics requirements

This study was approved by the Ethics in Research Committee at the Faculty of Dentistry, Ain Shams University, Egypt (approval number FDASU-ReclE121904).

## Conflict of interest

None disclosed.
